# Substaging and Stratified Treatment of T1 Bladder Cancer – a Nationwide Register-Based Study

**DOI:** 10.1016/j.euros.2026.06.011

**Published:** 2026-07-06

**Authors:** Ninna K. Nielsen, Peter B. Hjort, Erik B. Hansen, Charlotte Graugaard-Jensen, Jakob K. Jakobsen, Pernille S. Kingo, Jørgen B. Jensen

**Affiliations:** a Department of Clinical Medicine, Aarhus University, Aarhus, Denmark; b Department of Urology, Aarhus University Hospital, Aarhus, Denmark; c Department of Urology, Gødstrup Regional Hospital, Gødstrup, Denmark; d Greenland Centre for Health Research, Institute for Health and Nature, Ilisimatusarfik/University of Greenland, Nuuk, Greenland

**Keywords:** Bladder cancer, Non-muscle invasive bladder cancer, Pathological substaging, T1 substaging

## Abstract

**Background and objective:**

Substaging of T1 bladder cancer (BC) was implemented in Denmark in 2010 based on depth of lamina propria invasion—pT1a (superficial) and pT1b (deep)—with bladder-sparing therapy recommended for pT1a and early cystectomy for pT1b. This study evaluated outcomes of this substage-guided approach in a nationwide cohort.

**Methods:**

In this nationwide register-based study (2012–2018), all patients diagnosed with T1 BC were identified from the Danish Bladder Cancer Database. Patients were stratified by substage (pT1a, pT1b, unspecified pT1). Overall survival was estimated using Kaplan–Meier analysis; intravesical recurrence and progression were analysed using the Aalen-Johansen estimator, treating death as a competing risk; hazard ratios (HRs) were calculated using Cox models adjusted for sex, age, and comorbidities.

**Key findings and limitations:**

Among 3086 patients (pT1a: 1,542; pT1b: 1,195; unspecified pT1: 349), the 5-yr overall survival (OS) was 67% for pT1a, 53% for pT1b, and 53% for unspecified pT1 (*p* < 0.001). The 5-yr cumulative incidence of intravesical recurrence was 19% (95% CI: 17–22), 28% (95% CI: 24–32), and 29% (95% CI: 24–35), respectively (*p* < 0.001). Cause-specific HRs for recurrence and progression were significantly higher for pT1b and unspecified pT1 versus pT1a. As treatment was stratified by substage, outcomes reflect both tumour biology and management. Main limitations relate to the register-based design.

**Conclusions and clinical implications:**

Nationwide T1 substaging was feasible and retained prognostic value. pT1b disease was associated with worse outcomes, and one in five patients with pT1b who underwent cystectomy had muscle-invasive disease. These findings support substage-based management while warranting prospective evaluation accounting for treatment selection.

## Take Home Message

Pathological substaging of T1 bladder tumours was feasible to implement nationwide in Denmark. Patients with pT1b disease had worse survival, recurrence and progression outcomes than those with pT1a disease, reflecting the outcomes of a national substage-stratified management strategy.

ADVANCING PRACTICE **What does this study add?**

Our nationwide study provides real-world data from a complete seven-year cohort of Danish patients with tumour stage 1 (T1) bladder cancer. It demonstrates that pathological T1 substaging can be implemented at a nationwide level and identifies clinically meaningful differences in survival, recurrence, and progression between patients with pT1a and pT1b tumours. The findings reflect outcomes under a nationwide substage-stratified management strategy and support continued evaluation of this approach.

**Clinical Relevance**

Pathological substaging of T1 bladder cancer according to the depth of lamina propria invasion retains prognostic value when implemented in routine clinical practice. In this nationwide cohort, patients with pT1b tumors experienced worse survival and higher risks of recurrence and progression than those with pT1a disease, despite treatment stratification based on substage. These findings support the clinical utility of T1 substaging as a risk-stratification tool to guide individualized management and identify patients who may benefit from early radical treatment. Associate Editor: M. Carmen Mir, M.D; PhD.

**Patient Summary**

In this study, we examined a Danish nationwide cohort of patients with superficially invasive bladder cancer (stage T1). We found that dividing these patients into two groups based on tumour invasion depth helped predict the disease course. Patients with deeper tumours who do not undergo removal of their bladder have shorter survival and a higher risk of the cancer coming back, which supports treating the two groups differently.

## 1 Introduction

Worldwide, more than 600 000 bladder cancer (BC) cases are diagnosed annually, with non–muscle-invasive disease accounting for approximately 75%. Of these, T1 tumours—tumours invading the lamina propria but not the detrusor muscle—account for roughly 20% [1], [2].

T1 BC is a heterogeneous disease with substantial prognostic variability, leading to uncertainty regarding optimal treatment. European guidelines classify T1 BC with high-grade Ta tumours and recommend bladder-sparing management [3]. However, residual disease and under-staging at transurethral resection of bladder tumour (TURBT) [4], [5], and the risk of progression to muscle-invasive BC (MIBC) [6], [7], suggest this approach may be insufficient for some patients. Conversely, routine early cystectomy for all patients with T1 BC would overtreat many. This clinical dilemma emphasises the need for more precise risk-stratification tools to guide treatment decisions and achieve broader consensus on optimal management.

Depth of lamina propria invasion is associated with both progression and mortality [8]. A meta-analysis found that tumour infiltration depth was associated with disease progression, with a pooled hazard ratio (HR) of 3.29 (95% confidence interval [CI] = 2.39–4.51) [9].

Therefore, T1 substaging is considered prognostic [10], [11]. Multiple systems exist, but no consensus on the optimal approach has been reached.

The 2022 World Health Organization (WHO) classification recommends T1 substaging, advising pathologists to apply the system with which they are most familiar [12].

Since 2010, Danish national guidelines have recommended T1 BC substaging into pT1a or pT1b, depending on whether lamina propria invasion is superficial (close to the basal membrane) or deep (deeper, close to large vessels or the muscularis mucosa) [13]. Danish treatment guidelines recommend bladder-sparing treatment (TURBT, second resection and Bacillus Calmette–Guérin [BCG]) for pT1a tumours, whereas early cystectomy is recommended for pT1b tumours [13]. Cystectomy is also recommended in the following pT1a cases: presence of lymphovascular invasion, tumours larger than 3 cm, multifocal tumours, aggressive histological subtypes, recurrent pT1 tumour, if the tumour is palpable after endoscopic resection, or if a more advanced tumour is suspected on imaging. For patients who are unfit for cystectomy, radiotherapy may be considered.

### 1.1 Aim

This study aimed to evaluate the oncological outcomes of the Danish treatment approach with bladder-sparing therapy for pT1a tumours and early cystectomy for pT1b tumours.

## 2 Methods

### 2.1 Data sources

Data for this register-based cohort study were retrieved from the Danish Bladder Cancer Database (DaBlaCa-data), a national quality register including all patients diagnosed with BC in Denmark since 2010. DaBlaCa-data is based on data from the Danish National Patient Registry and the Danish Pathology Register (DPR).

In Denmark, all procedures and diagnoses in all patients managed at public hospitals and private clinics are registered in the Danish National Patient Registry using specific International Classification of Diseases, 10th Revision (ICD-10) codes [14]. All specimens and biopsies submitted for pathological assessment are registered in DPR using SNOMED codes for morphology, histological subtype, stage, and grade [15]. The algorithms behind DaBlaCa-data have been validated [16], [17].

The study was approved by the Regional Committee on Health Research Ethics for the Central Denmark Region (1-10-72-18-20) and registered on the internal list of research projects in the Central Denmark Region (1-16-02-537-18) approved by the Danish Data Protection Agency.

### 2.2 Study population

All Danish patients diagnosed with T1 BC between 2012 and 2018 were included. Only urothelial carcinomas, including subtypes, were included (SNOMED codes: M81203, M81213, M81223, M81303, M81313).

Patients who were registered as having received BCG treatment before their T1 diagnosis were excluded. Patients registered as having a ≥T2 diagnosis within 90 d of the first T1 diagnosis were not included in the dataset, as this was considered primary under-staging.

### 2.3 Second resections

DaBlaCa-data does not include information on second resections (repeat TURBTs). However, if a T1 substage higher than the initial substage was registered within 90 d, patients were reclassified accordingly.

### 2.4 Definitions

Patients were assigned to the treatment modalities “BCG”, “cystectomy”, or “radiotherapy” if they were registered as having received one of these within 120 d of the first T1 diagnosis. If they were registered with more than one of these modalities, the most radical treatment was registered. Cystectomy was considered more radical than radiotherapy, and radiotherapy more radical than BCG. If they received none of the abovementioned within 120 d, the treatment was registered as “TURBT only.” Information on systemic therapy was unavailable.

T1 recurrence was defined as a new T1 diagnosis in the DPR, at least 90 d after the initial T1 diagnosis. Progression was defined as a diagnosis of MIBC (≥T2) in the DPR at least 90 d after the initial T1 diagnosis.

### 2.5 Statistical analyses

For survival analyses, the index date was the date of the first T1 BC diagnosis. The study end date for censoring was 12 April 2024, the date of data extraction.

Overall survival (OS) was analysed using the Kaplan–Meier estimator, stratified by pathological substage (pT1a, pT1b, unspecified pT1), with group comparisons performed using the log-rank test. Survival time was calculated from the index date until death or censoring at the study end date. Patients who underwent early cystectomy and had ≥pT2 disease in their cystectomy specimen were excluded from survival analyses, as these cases were considered initial under-staging.

Cumulative incidence of intravesical T1 recurrence and progression to MIBC was estimated using the Aalen-Johansen estimator. In the recurrence analysis, progression and delayed cystectomy were counted as recurrence, and death was considered a competing risk. In the progression analysis, death and delayed cystectomy were treated as competing risks. Only patients with initial bladder-sparing treatment were analysed; that is, patients who underwent cystectomy as initial treatment were excluded.

Patients were followed from the index date until the first documented recurrence or progression, a competing event, or the end of the study. Differences between cumulative incidence functions for the three substages were evaluated using Gray’s test.

Absolute risks at 5 yr were extracted from the cumulative incidence function. Risk differences were calculated using the Newcombe hybrid score method. p values were calculated using the Wald test. Median follow-up among event-free patients was estimated using the reverse Kaplan–Meier method.

To estimate HRs, the Cox regression model was applied, adjusted for sex, age, and Charlson Comorbidity Index score, with pT1a as the reference group. The proportional hazards assumptions were evaluated using Schoenfeld residuals and log(-log) plots. p values were calculated using the Wald test.

### 2.6 Software

Data management and statistical analyses were performed using R statistical software, version 4.3.0 (21 April 2023).

## 3 Results

**Fig. 1:**
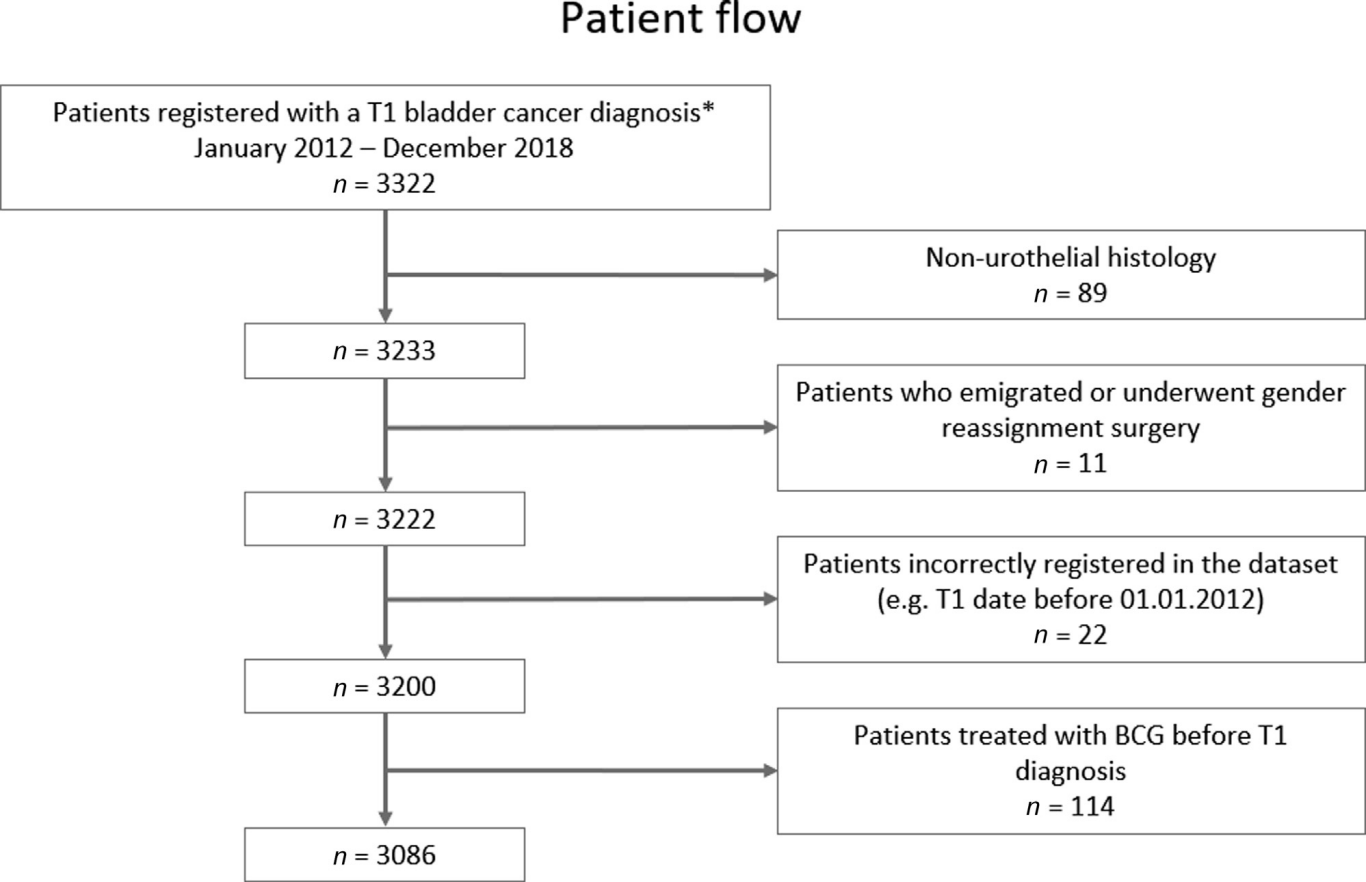
Flow diagram illustrating the derivation of the study cohort. *Patients registered with ≥T2 within 90 days of the first T1 diagnosis were not included in the study.

We identified 3086 patients diagnosed with T1 urothelial carcinoma between January 2012 and December 2018 (Fig. 1). Of these, 1542 (50.0%) patients were registered as pT1a, 1195 (39%) as pT1b, and 349 (11%) as unspecified pT1.

Within 120 d, 1449 (94%) patients with pT1a underwent bladder-sparing treatment, and 487 (41%) patients with pT1b underwent cystectomy, as per Danish guidelines.

Of the patients treated with cystectomy as initial treatment, 97/487 (20%) with pT1b had ≥ T2 disease in their cystectomy specimen, compared with 9/93 (9.7%) with pT1a. Patient characteristics are listed in Table 1.

**Table 1:** Patient characteristics by pathological T-stage at initial transurethral resection of bladder tumour

	Pathological T1 substage at TURBT		
	pT1a, *n* = 1542	pT1b, *n* = 1195	pT1 unspecified, *n* = 349
**Patient sex,*****n*****(%)**			
Male	1,208 (78%)	897 (75%)	265 (76%)
**Patient age, median (Q1, Q3)**	73 (67, 79)	73 (67, 80)	76 (69, 82)
**Charlson comorbidity score, median (Q1, Q3)**	1 (0, 2)	1 (0, 2)	1 (0, 2)
**Treatment,*****n*****(%)**			
BCG	833 (54%)	170 (14%)	136 (39%)
Cystectomy	93 (6.0%)	487 (41%)	33 (9.5%)
Radiotherapy	7 (0.5%)	86 (7.2%)	4 (1.1%)
TURBT only	609 (39%)	452 (38%)	176 (50%)
**Pathological T-stage at cystectomy,*****n*****(%)**			
pT1a, pT1b or pT1 unspecified	15 (16%)	70 (14%)	6 (18%)
≥ pT2	9 (9.7%)	97 (20%)	8 (24%)
pT0 or unknown	69 (74%)	320 (66%)	19 (58%)
**Death within 5 yr from diagnosis,*****n*****(%)**	508 (33%)	562 (47%)	164 (47%)

BCG = Bacillus Calmette–Guérin; TURBT = transurethral resection of bladder tumour.

Percentages are calculated within each substage.

We studied the distribution of substages across the regions of Denmark. In one region, 22% of patients were not substaged, while in the remaining regions, this proportion ranged from 4.5% to 14% (Supplementary Table 1). Overall, the proportion of patients with unspecified T1 declined over time, from 16% in 2012 to 6.9% in 2018.

**Fig. 2:**
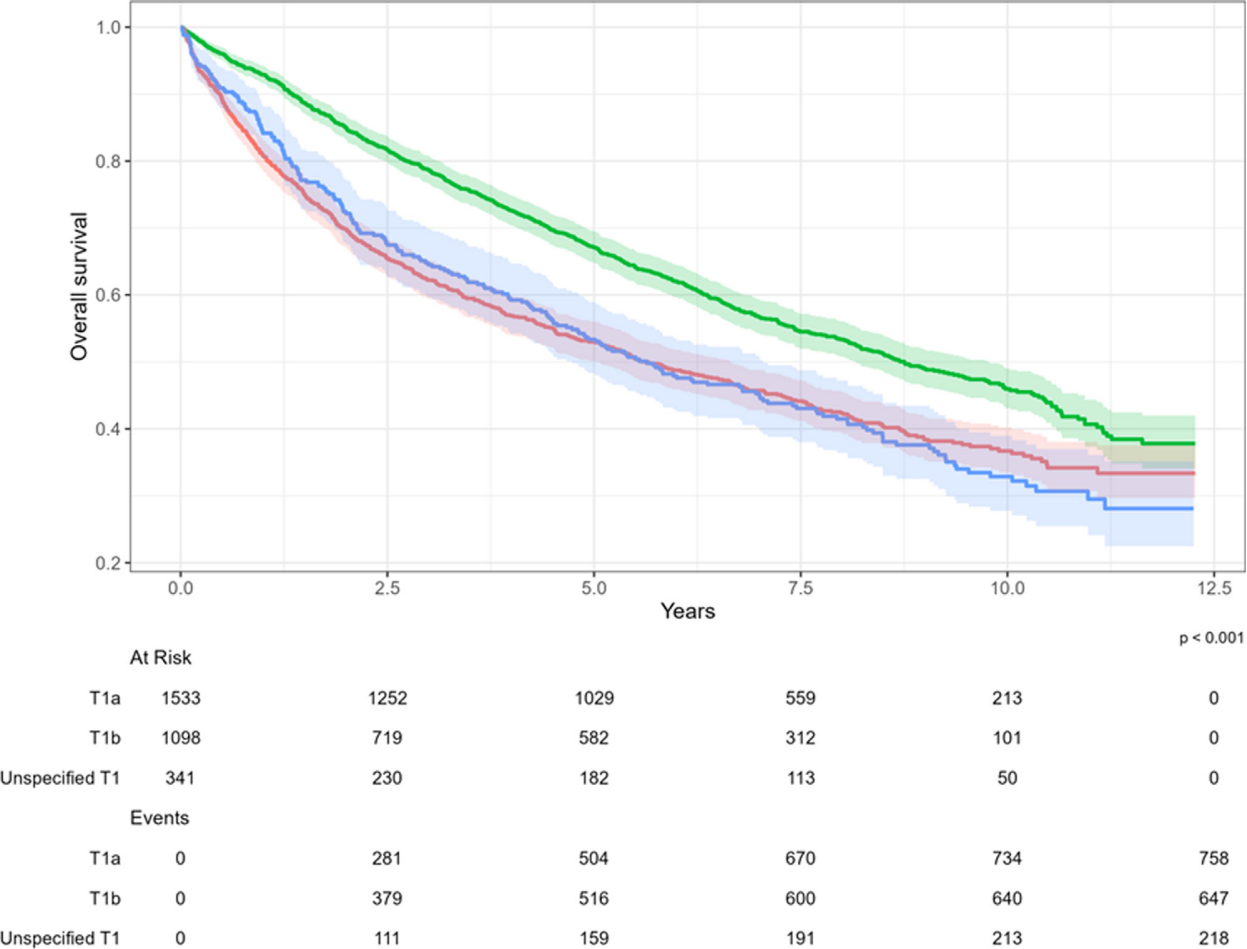
Overall survival, Kaplan-Meier estimate. Patients treated with early cystectomy and upstaged to MIBC in the cystectomy specimen are omitted. Green curve: pT1a, red curve: pT1b, blue curve: Unspecified pT1. Shaded areas: 95% confidence intervals.

Kaplan–Meier analysis demonstrated significant differences in OS across the three pathological substages (Fig. 2). At 5 yr, patients with pT1a had an estimated OS of 67% (95% CI = 64.8–69.5), whereas it was 53% for both pT1b (95% CI = 50.1–56.0) and unspecified pT1 (95% CI = 48.3–58.9). The log-rank test confirmed a statistically significant difference in survival between groups (*p* < 0.001).

As a sensitivity analysis, OS was re-estimated, including patients with ≥pT2 disease. Results were virtually identical to the primary analysis (Supplementary Table 2S).

**Fig. 3:**
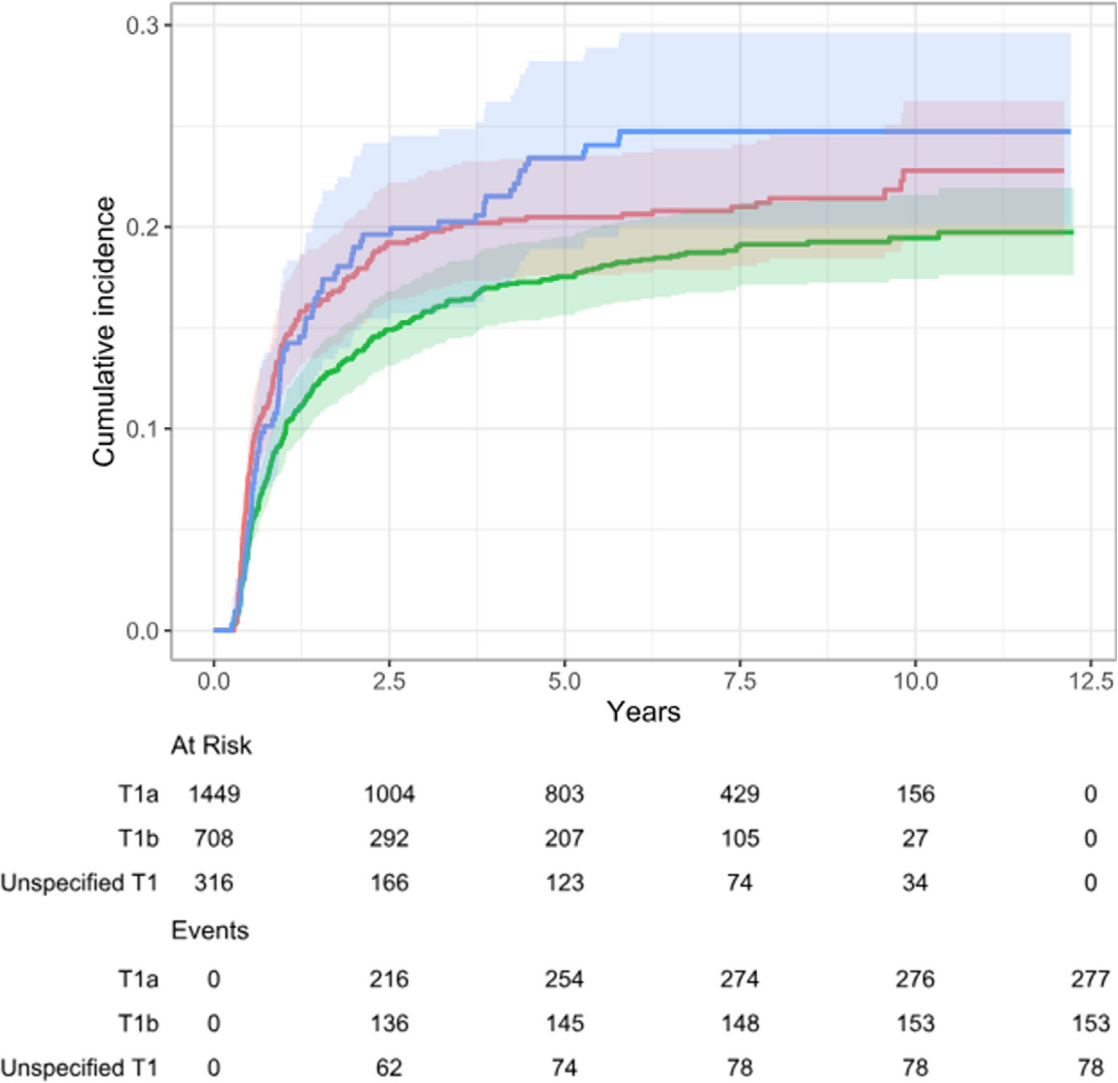

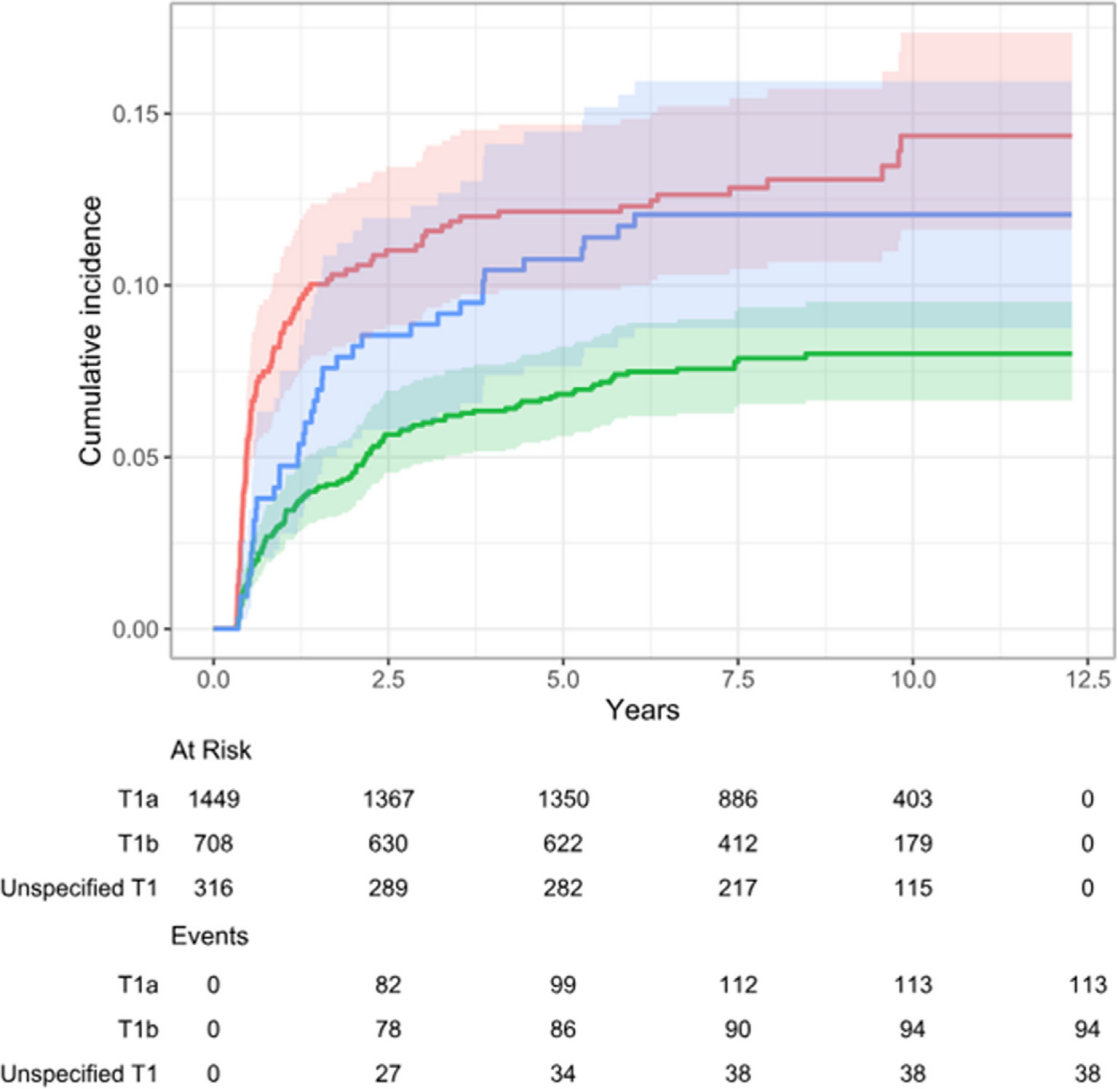
Top: Cumulative incidence of recurrence of T1 in bladder-preserved patients. Green curve: pT1a, red curve: pT1b, blue curve: Unspecified pT1. Shaded areas: 95% confidence intervals. Bottom: Cumulative incidence of progression to T2+ in bladder-preserved patients. Green curve: pT1a, red curve: pT1b, blue curve: Unspecified pT1. Shaded areas: 95% confidence intervals.

The cumulative incidence of T1 recurrence differed significantly across substages (Fig. 3A). At 5 yr, the cumulative incidence of recurrence was 19% (95% CI = 17–22) for pT1a, 28% (95% CI = 24–32) for pT1b, and 29% (95% CI = 24–35) for unspecified pT1 (Table 2). Gray’s test confirmed statistically significant differences between groups (*p* < 0.001). The cumulative incidence of progression to MIBC also differed among the T1 substages (Fig. 3B). At 5 yr, the cumulative incidence of progression was 6.8% (95% CI = 5.4–8.3) for pT1a, 11.9% (95% CI = 9.1–15.1) for pT1b, and 12.5% (95% CI = 8.5–17.2) for unspecified pT1 (Table 2), (*p* < 0.001).

**Table 2:** Risk of recurrence and progression in patients with preserved bladder

	Pathological T1 substage at TURBT		
	pT1a, *n* = 1449	pT1b, *n* = 708	pT1 unspecified, *n* = 316
**Death within 5 yr from diagnosis,** ***n*****(%)**	492 (34%)	444 (63%)	154 (49%)
**5-yr risk of T1 recurrence, % (95% CI)**	19% (17,22)	28% (24,32)	29% (24,35)
**Recurrence risk difference,** **pp (95% CI)**	–	+8.1 pp (3.6, 12.6), *p < 0.001	+9.9 pp (3.8, 16.2), *p = 0.002
**5-yr risk of progression to MIBC, % (95% CI)**	6.8% (5.4-8.3)	12% (9.1-15.1)	13% (8.5-17)
**Progression risk difference,** **pp (95% CI)**	–	+5.1 pp (1.9, 8.6), *p = 0.002	+5.7 pp (1.5, 10.6), *p = 0.014
**Cause-specific hazard ratio for T1** **recurrence, HR (95% CI)**	–	1.58 (1.30, 1.93), *p < 0.001	1.55 (1.20, 1.99), *p < 0.001
**Cause-specific hazard ratio for** **progression to MIBC, HR (95% CI)**	–	2.02 (1.46, 2.80), *p < 0.001	1.83 (1.22, 2.75), *p = 0.003

5-yr risk estimates from the cumulative incidence function and risk differences, with pT1a as reference. Cause-specific Cox regression analyses, adjusted for sex, age, and CCI score, with pT1a as reference.

CI = confidence interval, HR = hazard ratio, MIBC = muscle-invasive bladder cancer, TURBT = transurethral resection of bladder tumour. *Wald test.

Median follow-up among event-free patients was similar across substages. For OS, it was 8.7 yr (95% CI = 8.5–8.9) for pT1a, 8.5 yr (95% CI = 8.3–8.8) for pT1b, and 9.8 yr (95% CI = 9.2–10.1) for unspecified pT1. Corresponding values were 8.7 yr (95% CI = 8.5–8.9), 8.9 yr (95% CI = 8.5–9.3), and 9.9 yr (95% CI = 9.6–10.3) for recurrence-free survival, and 8.7 yr (95% CI = 8.5–9.0), 8.8 yr (95% CI = 8.4–9.2), and 9.9 yr (95% CI = 9.5–10.3) for progression-free survival.

Cause-specific HRs for both recurrence and progression were significantly higher for pT1b than for pT1a: 1.58 (95% CI = 1.30–1.93) (*p* < 0.001) and 2.57 (95% CI = 1.95–3.39) (*p* < 0.001), respectively. For unspecified pT1, cause-specific HRs were also significantly higher for both recurrence (HR = 1.55; 95% CI = 1.21–2.00; *p* < 0.001) and progression (HR = 1.90; 95% CI = 1.31–2.75; *p* < 0.001), compared with pT1a (Table 2).

## 4 Discussion

In this nationwide register-based study of Danish patients with T1 BC, pathological substaging was feasible at a nationwide level and was associated with clinically relevant differences in survival, recurrence, and progression.

Several studies have demonstrated the prognostic relevance of T1 bladder tumour substaging [9]. Our nationwide study supports these findings in a complete 7-yr cohort of patients with T1 BC. The low proportion of tumours that could not be substaged (11.3%) indicates that national implementation of T1 substaging is feasible. All Danish cases of T1 BC are discussed with or referred to cystectomy-performing centres, and all pT1 specimens undergo pathological review, supporting consistency and internal validity.

The substaging system applied in Denmark is based on depth of tumour invasion relative to the muscularis mucosa (above [pT1a], into [pT1b], or beyond [pT1c] this level), initially introduced in the 1990s [18] and subsequently simplified into the pT1a/pT1b two-tier system commonly used today [19]. Although identification of the muscularis mucosa can be inconsistent (15–83%) [20], potentially limiting reproducibility [21], multiple studies support the prognostic value of this histoanatomic approach [22], [23], [24], [25], [26]. Alternative substaging systems have been proposed [27], but this study presents the system used in Danish practice.

Our findings that pT1b disease was associated with higher hazards of recurrence and progression are consistent with previous meta-analytic evidence [9]. Recurrence and progression rates were lower than in prior studies [7], likely because recurrence was defined as T1 or higher; thus, Ta tumours were not registered as recurrences. Furthermore, 20% of patients undergoing cystectomy for pT1b disease had muscle-invasive pathology in their cystectomy specimen, which, in settings without early cystectomy, would likely be recorded as progression.

Importantly, management was guided by substage: most patients with pT1a disease received bladder-sparing therapy, whereas 41% of patients with pT1b disease underwent early cystectomy. The observed differences cannot be attributed to tumour biology alone, as the treatment strategy was determined by substage. This circularity is inherent to evaluating a guideline-directed strategy in routine practice and should be considered when interpreting the results. Accordingly, this study evaluates the real-world effectiveness of a substage-guided treatment strategy rather than the isolated biological effect of substage. In this context, treatment functions as a mediator of the clinical effect of substage, not a traditional confounder, and statistical adjustment would address a different causal question from our primary research question.

OS was lower in patients with pT1b and unspecified pT1 disease. Previously published estimates vary across cohorts, reflecting differences in treatment patterns and follow-up completeness. A recent US population-based study of T1 BC reported a 5-yr OS of 58%, where TURBT plus BCG was the recommended treatment regimen [28]. However, only a small proportion of patients were registered as having received BCG. In contrast, a European retrospective multicentre study reported a 10-yr all-cause mortality of 41.5% and a 5-yr OS of approximately 80% for patients with high-grade T1 disease treated with BCG, although substantial loss to follow-up may have partly inflated these estimates [29]. Another US register-based study reported a 5-yr OS of 56.0% for T1 BC overall, with markedly different estimates for cystectomy versus local treatment (71.1% vs 52.4%) [30]. Together, these data suggest that survival varies by treatment strategy and that selected patients with T1 disease may benefit from radical treatment. Other studies have shown a prognostic effect of T1 substaging on survival, with a French study identifying the pT1b substage as the strongest predictor of cancer-specific survival [26]. In our data, the cause of death was unavailable, precluding cancer-specific survival analyses. While cause-specific mortality would have provided additional specificity, OS remains a clinically meaningful end point in this elderly population with substantial competing mortality, as differences in cancer-specific mortality may not translate into overall survival benefit.

This study has limitations inherent to register-based research, including potential misclassification and missing information. However, the completeness of national follow-up and the consistently low proportion of unspecified cases suggest limited information bias. Although Danish registry reporting is generally reliable [14], [31], unmeasured confounding cannot be excluded. Variation in diagnostic thoroughness or coding practices across regions or time periods is also possible. The risk of systematic selection bias is mitigated by universal health care coverage and complete follow-up through the Danish Civil Registration System [32]. As shown in the Supplementary Table, the proportion of unspecified T1 cases remained low across regions, supporting consistent implementation of substaging.

The Cox model was adjusted only for sex, age, and Charlson Comorbidity Index. Important prognostic factors such as tumour grade, size, multiplicity, concurrent carcinoma in situ, lymphovascular invasion, smoking status, and detailed comorbidity burden were not available. These are established risk factors for recurrence and progression [3] and may have introduced residual confounding, affecting the HR estimates.

Unrecorded comorbidity or systemic therapy may have influenced treatment allocation, particularly among patients with pT1b disease managed conservatively, potentially contributing to higher mortality in patients with bladder preservation. The recurrence and progression analyses were restricted to patients who received initial bladder-sparing treatment, excluding the 41% of patients with pT1b disease who underwent early cystectomy. Patients with pT1b disease and bladder preservation represent a clinically distinct subgroup—most likely patients who were medically unfit for, or declined, cystectomy despite guideline recommendations. This selected subgroup may have poorer functional status and higher competing risks than the broader pT1b population, potentially overestimating the true difference in recurrence and progression rates between pT1b and pT1a. Accordingly, comparisons between substages in this analysis should be interpreted with caution.

Recurrence was defined as T1 or higher disease, as Ta recurrences were not available in the dataset. This definition lowers overall recurrence rates and limits external comparability.

## 5 Conclusion

In this nationwide study of T1 BC, pathological substaging was feasible to implement nationwide and retained clinically meaningful prognostic value in routine practice, identifying differences in survival, recurrence, and progression. Patients with pT1b disease had significantly worse outcomes than those with pT1a disease, and 1 in 5 patients with pT1b disease treated with cystectomy had muscle-invasive disease in the cystectomy specimen. Although treatment selection limits causal interpretation, these findings suggest that patients with T1 BC may warrant different management according to substage and indicate that pT1 substaging remains clinically relevant after implementation into national guidelines. Future prospective studies evaluating substage-guided management strategies while accounting for treatment selection and differences in patient characteristics are warranted.

  ***Author contributions:*** Ninna K. Nielsen had full access to all the data in the study and takes responsibility for the integrity of the data and the accuracy of the data analysis. The corresponding author certifies that the manuscript represents original and valid work and neither this manuscript nor one with substantially similar content under my authorship has been published or is being considered for publication elsewhere, except as described in an attachment, and copies of closely related manuscripts are provided. If requested, this corresponding author will provide the data or will cooperate fully in obtaining and providing the data on which the manuscript is based for examination by the editors or their assignees. All authors have agreed to allow the corresponding author to serve as the primary correspondent with the editorial office, to review the edited typescript and proof. Each author has given final approval of the submitted manuscript. Each author has participated sufficiently in the work to take public responsibility for all of the content. Each author qualifies for authorship by the contributions listed below:

*Administrative, technical, or material support*: None.

*Supervision*: Graugaard-Jensen, Jakobsen, Kingo, Jensen.

*Study concept and design*: Nielsen, Hansen, Jensen.

*Acquisition of data*: Hansen.

*Analysis and interpretation of data*: Nielsen, Hjort, Jensen.

*Drafting of the manuscript*: Nielsen.

*Critical revision of the manuscript for important intellectual content*: Hjort, Graugaard-Jensen, Jakobsen, Kingo, Jensen.

*Statistical analysis*: Nielsen, Hjort.

*Obtaining funding*: Nielsen, Jensen.

  ***Financial disclosures***: Ninna K. Nielsen certifies that all conflicts of interest, including specific financial interests and relationships and affiliations relevant to the subject matter or materials discussed in the manuscript (eg, employment/ affiliation, grants or funding, consultancies, honoraria, stock ownership or options, expert testimony, royalties, or patents filed, received, or pending), are the following: Jakob Kristian Jakobsen reports a research grant from the Novo Nordisk Foundation; travel support and a sponsored research agreement with Medac; and consultancy work for Cystotech. Jørgen Bjerggaard Jensen reports advisory board membership for Ferring, Roche, Cepheid, Urotech, Olympus, AMBU, Janssen, and Cystotech; speaker roles for Medac, Olympus, Photocure ASA, and Conmed; and research collaborations with Medac, Photocure ASA, Roche, Ferring, Olympus, Intuitive Surgery, Astellas, Cepheid, Nucleix, Urotech, Pfizer, AstraZeneca, MeqNordic, Laborie, OneMed, AMBU, and Cystotech. The remaining authors have nothing to disclose.

  ***Funding/Support and role of the sponsor*:** This study was completed as part of a PhD funded by Novo Nordisk Fonden Project Grant in Surgical Research (ref. NNF19OC0058534) and Nordic Cancer Union Research Grant (ref. A16003). The funding organisations had no role in the study design, conduct, data analysis or interpretation.

  ***Declaration of Generative AI and AI-assisted technologies in the writing process:*** During the preparation of this work, the first author used OpenAI ChatGPT-5 to improve readability and language. After using this tool, all authors reviewed and edited the content as needed and take full responsibility for the content of the publication.

## Appendix A Supplementary material

Supplementary Data 1 The supplementary material includes a table illustrating the substage distribution across the regions of Denmark and a sensitivity analysis of 5-year overall survival including patients who underwent early cystectomy and had ≥pT2 or higher in their cystectomy specimen.
