# Supplementary material for: Substaging and Stratified Treatment of T1 Bladder Cancer – a Nationwide Register-Based Study
Source: Eur Urol Open Sci. 2026 Jul 6;90:92–100. doi: 10.1016/j.euros.2026.06.011 (PMC13355817; doi:10.1016/j.euros.2026.06.011)
Supplement: Supplementary Data 1 — The supplementary material includes a table illustrating the substage distribution across the regions of Denmark and a sensitivity analysis of 5-year overall survival including patients who underwent early cystectomy and had ≥pT2 or higher in their cystectomy specimen. [file mmc1.docx]

| **Supplementary Table 2S. Sensitivity analysis on overall survival** | | | |
| --- | --- | --- | --- |
|  | **Original analysis^*^** | | |
|  | **pT1a**, n = 1,533 | **pT1b**, n = 1098 | **pT1 unspecified**, n = 341 |
| **Overall survival (95% CI)** | 67.06% (64.75-69.44) | 52.97% (50.21-55.88) | 53.00% (48.02-58.51%) |
|  |  |  |  |
|  | **Sensitivity analysis^†^** | | |
|  | **pT1a**, n = 1,542 | **pT1b**, n = 1195 | **pT1 unspecified**, n = 349 |
| **Overall survival (95% CI)** | 67.12% (64.81-69.52) | 53.00% (50.13-56.04) | 53.37% (48.33-58.94) |
| 5-year overall survival calculated using the Kaplan Meier-estimator. ^*^Original analysis: Patients who underwent early cystectomy and had ≥pT2 or higher in their cystectomy specimen were excluded, as these cases were considered to reflect initial understaging. ^†^Sensitivity analysis: Including patients who underwent early cystectomy and had ≥pT2 or higher in their cystectomy specimen. | | | |

| **Supplementary Table 1S. Patient distribution across the regions of Denmark.** | | | |
| --- | --- | --- | --- |
|  | **TURBT T-stage** | | |
|  | **pT1a**, n = 1,542 | **pT1b**, n = 1,195 | **pT1 unspecified**, n = 349 |
| **Region, n (%)** |  |  |  |
| North Denmark Region, n = 458 | 240 (52%) | 117 (26%) | 101 (22%) |
| Central Denmark Region, n = 624 | 331 (53%) | 265 (42%) | 28 (4.5%) |
| Region of Southern Denmark, n = 696 | 307 (44%) | 295 (42%) | 94 (14%) |
| Capital Region of Denmark, n = 844 | 405 (48%) | 356 (42%) | 83 (9.8%) |
| Region Zealand, n = 462 | 258 (56%) | 162 (35%) | 42 (9.1%) |
| Private hospitals, n = 2 | 1 (50%) | 0 (0%) | 1 (50%) |
| Patient distribution across the regions of Denmark. Percentages are calculated within each region. | | | |
